# Optimize the parameters for the synthesis by the ionic gelation technique, purification, and freeze-drying of chitosan-sodium tripolyphosphate nanoparticles for biomedical purposes

**DOI:** 10.1186/s13036-024-00403-w

**Published:** 2024-01-25

**Authors:** Stephany Celeste Gutiérrez-Ruíz, Hernán Cortes, Maykel González-Torres, Zainab M. Almarhoon, Eda Sönmez Gürer, Javad Sharifi-Rad, Gerardo Leyva-Gómez

**Affiliations:** 1 Departamento de Farmacia, Facultad de Química, Universidad Nacional Autónoma de México, Ciudad de México, Mexico; 2 Departamento de Genómica, Laboratorio de Medicina Genómica, Instituto Nacional de Rehabilitación Luis Guillermo Ibarra Ibarra, Ciudad de México, Mexico; 3 CONACyT-Laboratorio de Biotecnología, Instituto Nacional de Rehabilitación Luis Guillermo Ibarra Ibarra, Ciudad de México, 14389 Mexico; 4 Department of Chemistry, College of Science, King Saud University, P. O. Box 2455, Riyadh, 11451 Saudi Arabia; 5 Department of Pharmacognosy, Faculty of Pharmacy, Sivas Cumhuriyet University, Sivas, Turkey; 6 Facultad de Medicina, Universidad del Azuay, Cuenca, Ecuador

**Keywords:** Chitosan, Freeze-drying, Nanoparticles, Optimization, Purification, Tripolyphosphate

## Abstract

**Background:**

Polymeric nanoparticles can be used for wound closure and therapeutic compound delivery, among other biomedical applications. Although there are several nanoparticle obtention methods, it is crucial to know the adequate parameters to achieve better results. Therefore, the objective of this study was to optimize the parameters for the synthesis, purification, and freeze-drying of chitosan nanoparticles. We evaluated the conditions of agitation speed, anion addition time, solution pH, and chitosan and sodium tripolyphosphate concentration.

**Results:**

Chitosan nanoparticles presented an average particle size of 172.8 ± 3.937 nm, PDI of 0.166 ± 0.008, and zeta potential of 25.00 ± 0.79 mV, at the concentration of 0.1% sodium tripolyphosphate and chitosan (pH 5.5), with a dripping time of 2 min at 500 rpm. The most representative factor during nanoparticle fabrication was the pH of the chitosan solution, generating significant changes in particle size and polydispersity index. The observed behavior is attributed to the possible excess of sodium tripolyphosphate during synthesis. We added the surfactants poloxamer 188 and polysorbate 80 to evaluate the stability improvement during purification (centrifugation or dialysis). These surfactants decreased coalescence between nanoparticles, especially during purification. The centrifugation increased the zeta potential to 40.8–56.2 mV values, while the dialyzed samples led to smaller particle sizes (152–184 nm). Finally, freeze-drying of the chitosan nanoparticles proceeded using two cryoprotectants, trehalose and sucrose. Both adequately protected the system during the process, and the sugar concentration depended on the purification process.

**Conclusions:**

In Conclusion, we must consider each surfactant's benefits in formulations for selecting the most suitable. Also, it is necessary to do more studies with the molecule to load. At the same time, the use of sucrose and trehalose generates adequate protection against the freeze-drying process, even at a 5% w/v concentration. However, adjusting the percentage concentration by weight must be made to work with the CS-TPP NPs purified by dialysis.

## Introduction

The development of smart polymeric materials has been one strategy implemented for delivering and releasing drugs with various biomedical purposes, including wound healing and antimicrobial action [1–4]. Nanoparticles (NPs) can be used efficiently due to their surface-to-volume ratio while reducing the distance from the delivery point to the growing cells [5, 6]. Furthermore, the need to effectively deliver biomolecules, such as antimicrobial agents, growth factors, and genes, can be met with the help of NPs since these polymeric devices protect from degradation by proteases and release in a controlled manner to reduce the frequency of administration [7].

Chitosan (CS) is a linear polysaccharide derived from chitin, which can be obtained from crustacean shells, and is composed of glucosamine and N -acetylglucosamine subunits linked by [1–4] glycosidic bonds. It is a hydrophilic biopolymer characterized by high biocompatibility and biodegradability, non-toxicity, biological adhesiveness, and hemostatic effect, besides being one of the few polymers with antibacterial properties. CS can modulate platelet activation during wound healing and promote blood coagulation in the hemostasis phase. Likewise, CS can regulate the release of pro-inflammatory factors and the activity of inflammatory cells in the inflammatory phase, providing a favorable microenvironment for wound healing. Furthermore, CS acts as non-protein matrix support for tissue growth in the proliferative process.

In addition, CS gradually depolymerizes to release N-acetyl-β-d-glucosamine, which stimulates fibroblast proliferation, hyaluronic acid synthesis, angiogenesis, and collagen deposition at the wound site. Such events enhance the wound-healing process and prevent scar formation in the remodeling process [8, 9]. These characteristics make highly attractive the development of CS-based systems for various biomedical devices for chronic conditions and routes of administration [10–12]. However, it is essential to note that the properties of CS are highly dependent on the microenvironment's pH, the polymer's molecular weight, the concentration, and the degree of deacetylation, which influence its physicochemical properties. The pKa of the primary amine of CS is around 6.5, depending on the degree of N-deacetylation [13]. This group also contributes to the solubility of CS, presenting a good solubility in dilute organic acids with a pH below 6.5. In addition to solubility, the protonated amine contributes to the antibacterial and mucoadhesive properties of CS [14].

Moreover to its affinity for metals, proteins, and dyes, CS can also form complexes with anions, such as sulfate and phosphate, due to its cationic nature [8]. These characteristics can be well exploited to elaborate CS- sodium tripolyphosphate (TPP) NPs by the ionic gelation technique. This technique involves the complexation between positively and negatively charged species upon mechanical agitation, separating CS into spherical particles of different sizes and surface charges. The ionic gelation technique is often attractive due to its mild processing needs, involving an aqueous environment with low toxicity and simple conditions for encapsulation of the drug [13].

Although the ionic gelation method is relatively simple to prepare and execute, achieving the desired size and uniformity can pose difficulties [15]. The mucoadhesive properties of chitosan in conventional NPs synthesis often lead to the formation of large particles or aggregates of smaller ones [16]. The physical characteristics and stability of nanoparticle formation are influenced by factors such as pH, solute concentration, surfactant addition, flow rate, and stirring speed, as well as the inclusion of purification and freeze-drying steps [15–18].

A purification process is necessary, as the composition of colloidal nanomaterials not only impacts their transport and distribution in the body but also plays a crucial role in toxicity issues. Therefore, it is essential to ensure the acquisition of a safe nanosystem, free of impurities and raw materials [19]. Although centrifugation is a common technique for this purpose, the problem of resuspension and the loss of structure and uniformity of CS NPs after this procedure is recognized [15, 20]. Consequently, various strategies have been adopted to address this challenge.

It is crucial to preserve the suspension characteristics of the nanoparticulate systems because the lack of stability of the colloid may restrict its application for biomedical purposes. In this respect, it is well known that the polymeric matrix increases in size and fractures in a few weeks, depending on the storage conditions [21, 22]. This process of swelling and aggregation over time can be attributed to Brownian motion and osmosis due to the presence of TPP [22]. Due to this, the NPs stored as an aqueous suspension undergo solubilization and/or degradation of the polymers, and drug leakage or desorption often occurs [23]. The freeze-drying process is the most popular technology to remove moisture and preserve nanoparticulate systems for a long time and overcome this lack of physicochemical stability. However, the high susceptibility of CS to environmental factors and process conditions, such as freezing, can impose stress on its structure and cause polymer degradation [12]. In some cases, it may even be difficult to achieve complete redispersion after freeze-drying due to aggregation or irreversible melting of the NPs [23]. Thus, the addition of a suitable cryoprotectant at optimal concentrations prior to freezing is necessary, which is formulation specific for polyelectrolytes [24, 25].

Although several methods have been described for obtaining CS NPs for various purposes, it is essential to carefully adjust the parameters of synthesis, purification, and preservation to achieve optimal properties applicable in the medical field. In this context, the primary focus of this work was to study the effects of pH, CS concentration, TPP concentration, surfactant addition, stirring speed, and drop flow rate to optimize the synthesis of CS-TPP NPs under mild conditions using the ionic gelation technique. We evaluated and analyzed the role of the formulation after implementing two purification strategies (centrifugation and dialysis), as well as the effect of certain cryoprotectants during freeze-drying to reduce aggregation and particle overgrowth.

## Materials and methods

### Materials

Low molecular weight CS with 75–85% deacetylation (Sigma Aldrich), TPP (Sigma Aldrich), polysorbate 80 (P80; Drogueria Cosmopolita), poloxamer 188 (P188; Sigma Aldrich), and 98% sodium hydroxide (Sigma Aldrich) were used to prepare the CS-TPP NPs. Anhydrous glycerol (Sigma Aldrich) and 50 KDa dialysis bags (Spectra/Por® 6) were used during purification. Cryoprotectants used during freeze-drying were sucrose ≥ 99.5% (Sigma Aldrich) and trehalose dihydrate (Sigma Aldrich).

### Preparation of CS-TPP NPs

We prepared the CS-TPP NPs by the ionic gelation method, using diluted acetic acid and TPP as the polyanion. First, 5 mL of low molecular weight CS solution was dissolved in 1% v/v acetic acid, with pH adjustment using 20% sodium hydroxide solution; subsequently passed through a 0.22 μm filter. Stirring continued and was followed by adding 1.7 mL of TPP at room temperature. Finally, the mix was stirred for 30 min. The evaluated values of CS and TPP concentration, as well as those related to pH, flow rate, and flow velocity, are described in the following section (Optimization of parameters for synthesis).

### Optimization of parameters for synthesis

The lower and upper limits of the selected values were based on literature data regarding the solubility of CS and the synthesis conditions of CS-TPP NPs through ionic gelation under characteristics similar to those of the CS used [26–30].

Formulation optimization occurred in two steps. Initially, we conducted a complete 3^3^ factorial experimental design to evaluate the following independent factors: pH (4.6, 5.0, and 5.5 ± 0.05), concentrations of CS and TPP (0.1%, 0.3%, and 0.5% w/v), with a drip time set at 4 min (0.43 mL/min) and an agitation speed of 600 rpm. This resulted in 27 different formulations.

Subsequently, a two-level design was embedded to evaluate the impact of drip time at 2 and 6 min (with flow rates of 0.85 mL/min and 0.28 mL/min, respectively) at different agitation speeds (500 rpm and 700 rpm), with pH variations (4.6 and 5.5 ± 0.05). We performed this under fixed concentrations of CS and TPP (0.1% in both cases), determined based on the outcomes from the initial phase of the optimization (refer to the Results section). Consequently, we established a total of 8 distinct synthesis conditions.

The recorded dependent values included particle size, polydispersity index (PDI), and zeta potential.

### Evaluation of surfactant addition in the formulation

We evaluated the stabilizing effect of the surfactants P80 and P188 in the formulation at a concentration of 1% w/v in both cases. These surfactants were added separately during the solubilization of CS in acetic acid. The preparation of CS-TPP NPs followed the same procedure as mentioned above.

### Purification of CS-TPP NPs

After the fabrication of CS-TPP NPs, purification proceeded with the evaluation of two methods: centrifugation and dialysis.

For the centrifugation method, 1.5 mL of the samples were placed in Eppendorf tubes at 15000 rpm (21885xg) for 2 to 3 h. After that, the pellet was resuspended in deionized water with the help of a vortex for 30 s. Also, we evaluated the influence of the presence of glycerol bedding during centrifugation in amounts of 10, 30, and 50 μg.

For the dialysis method, a 50 kDa molecular weight cut-off bag in 150 mL of distilled water was used for each mL of sample. The methodology was performed at room temperature at 130 rpm, and the samples were taken at 3, 5, and 21 h. There were two medium changes before the first sampling (45 min and 2 h), one before the second (4 h), and the third (7 h).

### Characterization of CS-TPP NPs

Particle size analysis, PDI, and the samples' zeta potential measurements were registered using a Malvern Zetasizer Nano Series instrument to characterize the samples before and after the purification process. The solutions were loaded into a disposable cell, diluted in deionized water, and the analysis was carried out at 25 °C using a dispersion angle of 173°. We employed a disposable folded capillary tube to analyze the zeta potential. The prepared solutions were measured three times per sample, and the results were reported as mean particle size ± SD, mean PDI ± SD, and mean zeta potential ± SD.

CS-TPP NPs size and geometry were analyzed by scanning electron microscopy (SEM). Briefly, we obtained the dispersion of CS-TPP NPs by dialyzing the samples with P188 and then diluting it to 1:10 with distilled water. We placed a drop on a coverslip and allowed it to dry at room temperature. After fixing the coverslip with carbon tape, we applied a thin layer of gold using the JOEL Fine Coat Ion JFC-1100 for plasma assisted deposition. Finally, we analyzed the sample with a SEM (CROSS-BEAM 550, ZEISS) using an acceleration voltage of 5 kV and magnifications of 5.06 and 14.75 kX.

### Yield in the manufacture

CS-TPP NPs with P80 and P188 were accurately collected and weighed after freeze-drying. The % yield was then determined using the formula presented below:

$$\%\;{\text{yield}}=\frac{\text{NPsW}}{\textrm{TW}}\;\times\;100$$

Where NPsW is the weight of dry CS-TPP NPs and TW is the total weight of solids used to synthesize the CS-TPP NPs.

### Freeze-drying of CS-TPP NPs

Following the purification of the CS-TPP NPs, we assessed the protective effect of trehalose and sucrose to prevent coalescence during freeze-drying. Concentrations of 5% and 10% w/v of each sugar were added separately. Samples were frozen at -60 °C for 3 h and freeze-dried for 24 h under reduced pressure conditions (< 0.1 mbar) using a SCIENTZ-10N freeze-dryer.

### Statistical analysis

Statistical analysis was performed with the STATGRAPHICS® centurion XV program. To evaluate the studied factors' effects, we used variance analysis (ANOVA). Moreover, we presented the interactions between the factors, the means of the treatments, and the minimum values of significant difference (*p* < 0.05). Confidence intervals resolved the difference between pairs of means using Tuckey's test. The significance level was set at *p* < 0.05.

## Results

### Optimization of parameters for synthesis

We optimized the formulation by analyzing the impact of the independent variables on the responses using a design of experiments [31]. The results of full factorial experimental design 3^3^ indicated that the most suitable concentration of CS and TPP is 0.1% w/v, in both cases, for the three pH levels determined by the lower PDI, suggesting a higher monodispersity. Table 1 represents the average sizes and PDI for the 27 formulations. PDI values less than 0.2 for CS NPs are exceptional in the literature.

**Table 1:** Particle size and PDI of the factorial design, under the influence of CS and TPP concentration and pH

CS concentration (%w/v)	TPP concentration (%w/v)	pH	Particle size (nm)	PDI	Appearance
0.1	0.1	4.6	112.9 ± 1.9	0.220 ± 0.017	Clear
		5.0	150.2 ± 3.6	0.182 ± 0.007	Clear
		5.5	197.2 ± 2.9	0.172 ± 0.019	Opalescent
	0.3	4.6	> 1000	0.688 ± 0.263	Clear
		5.0	> 1000	0.721 ± 0.295	Clear
		5.5	> 1000	0.883 ± 0.210	Agglomerates
	0.5	4.6	> 1000	0.653 ± 0.285	Clear
		5.0	> 1000	0.460 ± 0.221	Agglomerates
		5.5	> 1000	0.857 ± 0.140	Agglomerates
0.3	0.1	4.6	248.3 ± 8.2	0.388 ± 0.090	Clear
		5.0	253.4 ± 7.7	0.313 ± 0.035	Clear
		5.5	608.5 ± 81.3	0.328 ± 0.088	Clear
	0.3	4.6	280.5 ± 7.7	0.501 ± 0.022	Clear
		5.0	> 1000	0.422 ± 0.313	Opalescent
		5.5	> 1000	0.703 ± 0.128	Agglomerates
	0.5	4.6	> 1000	0.426 ± 0.244	Opalescent
		5.0	> 1000	0.820 ± 0.298	Opalescent
		5.5	> 1000	0.627 ± 0.259	Agglomerates
0.5	0.1	4.6	456.4 ± 75.5	0.828 ± 0.166	Gelling
		5.0	> 1000	0.868 ± 0.166	Gelling
		5.5	> 1000	0.397 ± 0.159	Agglomerates
	0.3	4.6	704.0 ± 182.7	0.808 ± 0.109	Clear
		5.0	427.0 ± 56.3	0.648 ± 0.094	Opalescent
		5.5	> 1000	0.859 ± 0.151	Agglomerates
	0.5	4.6	722.4 ± 121.7	0.930 ± 0.080	Opalescent
		5.0	615.7 ± 62.3	0.900 ± 0.092	Opalescent
		5.5	> 1000	0.681 ± 0.144	Agglomerates

CS: TPP 3:1; *n* = 2

We determined the particle size, PDI, and zeta potential for the three pH levels (Table 2) under 0.1% concentration of CS and TPP. A decrease in the size of the NPs and an increase in the PDI and zeta potential were observed as the pH of the medium decreased.

**Table 2:** Particle size, PDI, and Z-potential at different pH of CS-TPP NPs synthesis

pH	Particle size (nm)	PDI	Zeta potential (mV)
4.6	110.4 ± 2.158	0.245 ± 0.028	34.90 ± 2.26
5.0	122.1 ± 3.253	0.219 ± 0.013	29.83 ± 0.83
5.5	172.8 ± 3.937	0.166 ± 0.008	25.00 ± 0.79

*n* = 3; mean ± SD

Subsequently, we determined particle size and PDI through a new two-level experimental design, specifically focusing on pH evaluation under mild agitation conditions and short synthesis times. This approach was directed towards potential future applications of the system with sensitive molecules commonly used in biomedical contexts. Following this, we adjusted the results according to the generated model, and the ANOVA test confirmed its suitability. Both particle size and PDI exhibited statistically significant differences concerning pH and drip time.

The polynomial equation generated for particle size is presented below in terms of coded factors:

$$\text{Particle size}\ =\ -148.531\ +\ 0.0754074\text{A}\ +\ 50.6481\text{B}-54.9213\text{C}-0.00740741\text{AB}-0.00991667\text{AC}\ +\ 13.3796\text{BC}$$

**Fig. 1:**
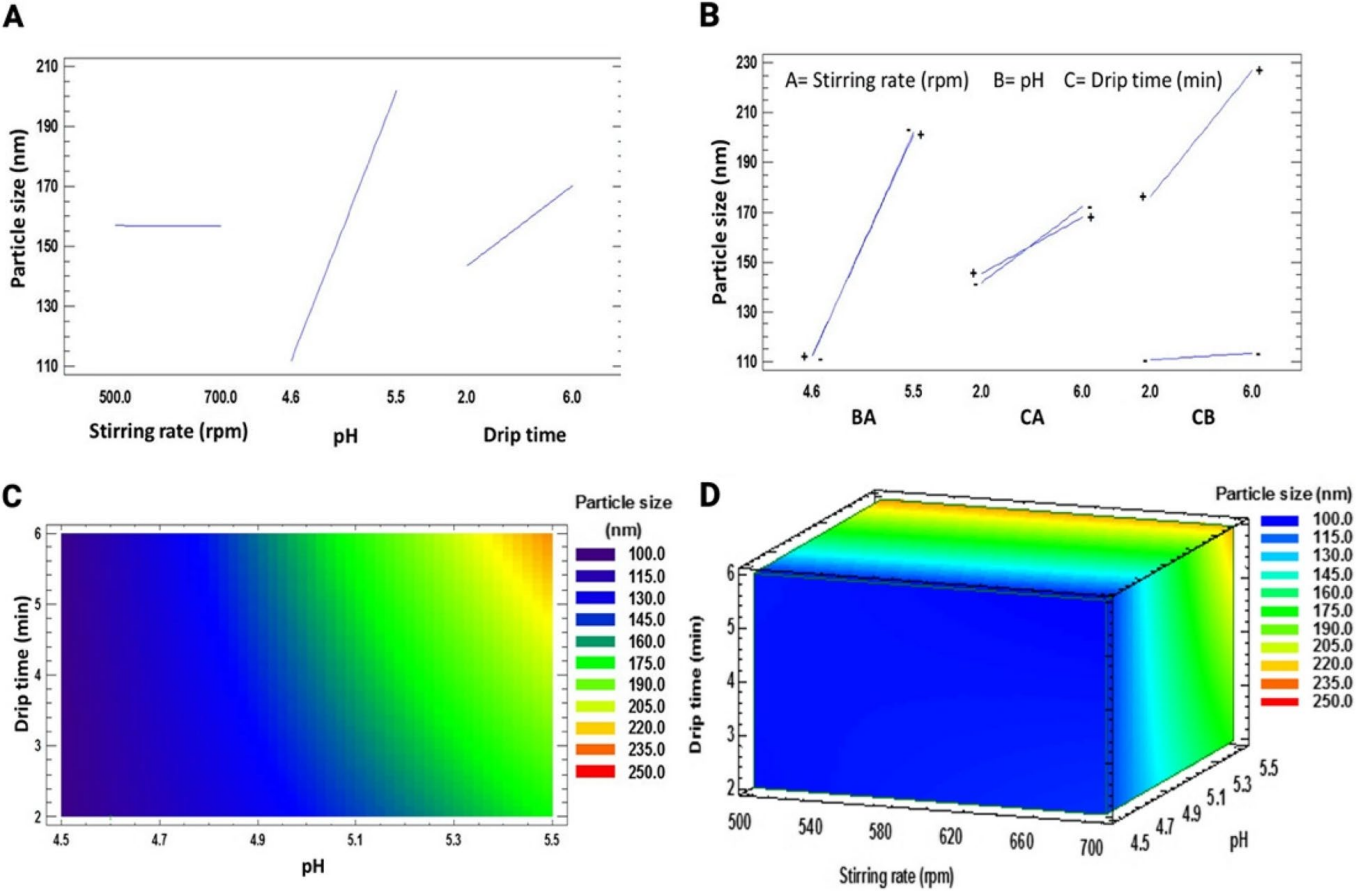
**A** Main effects plot for particle size, (**B**) Interactions plot for particle size, (**C**) Contour plot showing the effect of drip time vs. pH at 500 rpm for particle size, and (**D**) 3-D contour plot representing the effect of drip time, pH, and stirring rate

Where A is the stirring rate, B is the pH, and C is the drip time. This equation shows that agitation speed and pH positively affect particle size, which means that the particle size will increase at a higher level of the two factors. On the other hand, the dripping time has a negative effect and, therefore, reduces the particle size as the time in which the TPP is dripped increases. However, the most pronounced effects correspond to pH and drip time, as indicated in the equation and Fig. 1A, which corresponds to the individual effect of independent factors on particle size. When the agitation speed was modified, there were no significant changes, so a horizontal line appeared, with no pronounced difference in the slope when the factor level was changed.

The interactions between drip time and agitation speed and between drip time and pH showed statistically significant differences. The latter has a larger effect (Fig. 1B). The interaction between the independent factors will affect the final response, which is best predicted using contour plots and response surface [32] (Fig. 1C and D). From these plots, it can be deduced that the smallest particle size will be obtained when working with the lowest drip time and pH levels.

During the optimization of the formulation, another objective was to obtain a monodisperse system, which is ideal for its application for medical purposes. Therefore, the polynomial equation for the PDI was obtained:

$$\text{PDI }= 0.963352 - 0.000857222*\text{A }- 0.155185*\text{B }+ 0.0220833*\text{C }+ 0.000172222*\text{AB }- 0.0000104167*\text{AC }- 0.00166667*{\text{BC}}$$

**Fig. 2:**
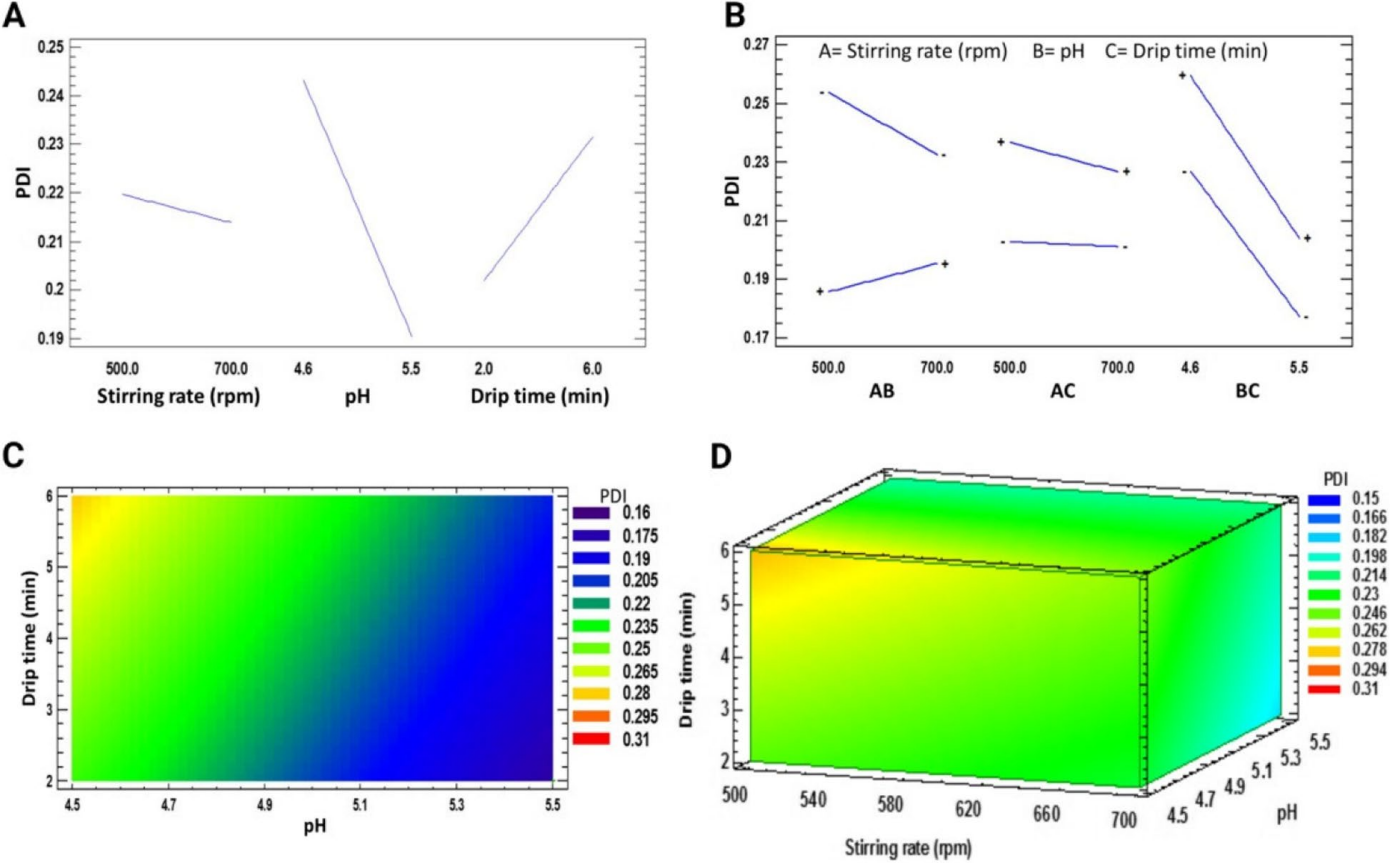
**A﻿** Main effects plot for PDI, (**B**) Interactions plot for PDI, (**C**) Contour plot of drip time vs. pH at 500 rpm for PDI, (**D**) 3-D contour plot representing the effect of drip time, pH, and stirring rate

Where A is the stirring rate, B is the pH, and C is the drip time. It was found from the equation that the first two factors have a negative effect, generating a decrease in the PDI when working with the highest levels. The individual effects of both pH and drip time presented significant differences in the slopes shown when changing the value of the independent factors on the PDI (Fig. 2A). The pH had a more substantial impact on the decrease in polydispersity.

The interaction between agitation speed and pH generated the most remarkable difference in PDI, presenting a decrease in the effect when working with the low value of pH and the high value of agitation speed. The contrary effect on PDI occurred when working with the opposite values of the factors (Fig. 2B). However, the interaction that presented the most significant decrease in effect was between pH and drip time. Therefore, the optimization is best seen in the contour and response surface plots (Fig. 2C and D). We concluded that the lower drip time and higher pH generate the lowest PDI values.

The goodness of fit to the linear regression model was satisfactory for both variables, as shown in Table 3. As a result, we found that the lowest PDI is generated from the highest pH level; however, it is essential to consider that this will increase particle size.

**Table 3:** Significance of the factors studied on the response variables

Variable	Model order	Lack of adjustment (*p*-value)	R^2^	R^2^ adjusted	Optimal values (goal: minimize)
Particle size	2°	0.0782	99.50	99.40	Stirring rate = 500 rpm
					Drip time = 2 min
					pH = 4.6
PDI	2°	0.0952	93.13	92.10	Stirring rate = 500 rpm
					Drip time = 2 min
					pH = 5.5

The pH changed the appearance of the NPs suspension during the synthesis, from translucent to opalescent when the pH value increased.

The variation of the studied parameters reveals the strong influence of pH and ionic interactions in the system. This is highly relevant for biomedical applications, as pH and ionic strength can vary between tissues and organs during pathogenesis and disease progression [33]. Such considerations allow the prediction of the drug delivery profile in different microenvironments [34] and conditions that enhance the antimicrobial properties that characterize the biopolymer [30, 35]. Even in more novel systems, CS has been used as a smart indicator of pH changes [36]. Another critical aspect to consider is that this type of system is sensitive to the exchange of ions present in the medium, which can affect the stability and durability of the material in different body fluids [37].

### Purification of CS-TPP NPs

Although several methods exist to purify colloidal nanomaterials, we chose centrifugation and dialysis to separate CS-TPP NPs from waste that did not form the particle. We also evaluated the glycerol bed's presence during the centrifugation technique, during the procedure, and during the centrifugation time.

**Fig. 3:**
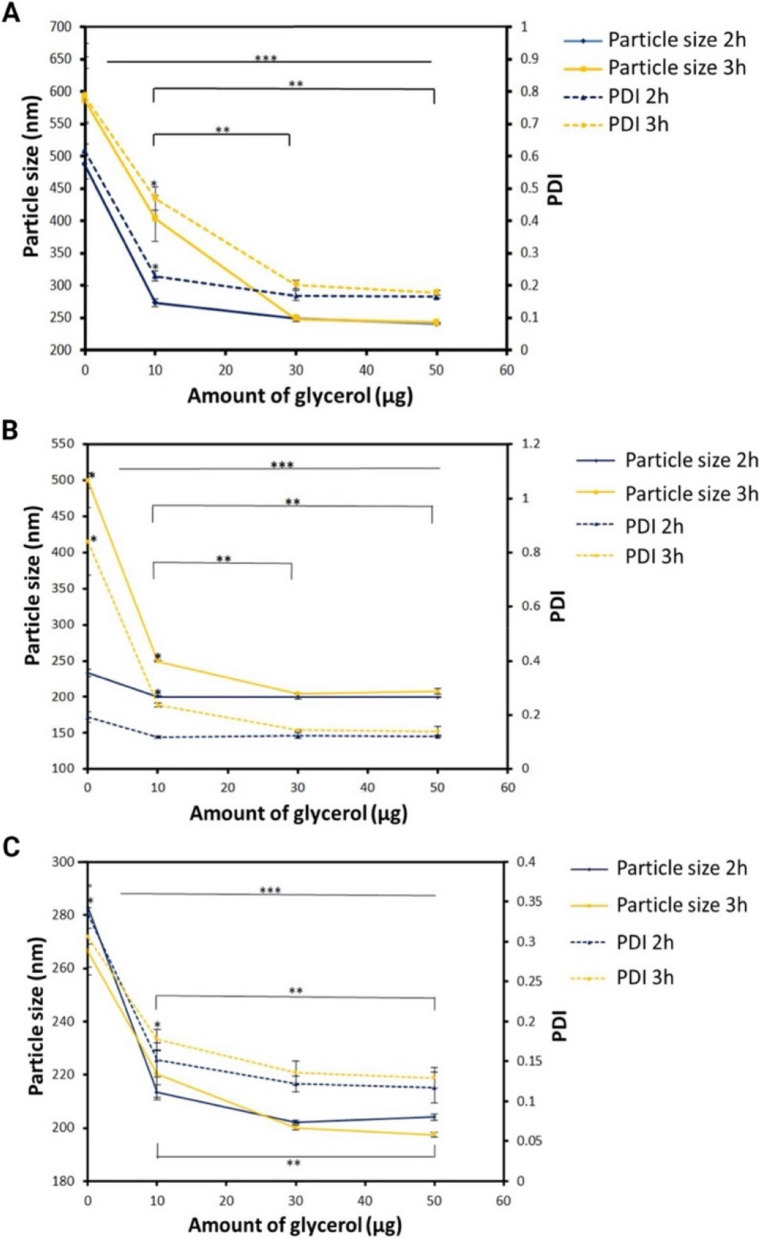
Particle size and PDI of the samples as a function of centrifugation time (2 and 3 h) and amount of glycerol (0, 10, 30, and 50 μg). **A** CS-TPP NPs without surfactant, (**B**) CS-TPP NPs with P80, and (**C**) CS-TPP NPs with P188. *n* = 3; two-way ANOVA, Tuckey post hoc test. * *p* < 0.05 between times, ** *p* < 0.05 between amount of glycerol, *** *p* < 0.05 between total points studied vs. control. Control = sample before centrifugation

Figure 3 demonstrates the glycerol effect for CS-TPP NPs with and without surfactants. Increasing the amount of glycerol up to 30 μg decreases the average particle size and PDI, with no significant difference when glycerol increased to 50 μg. Consequently, we established the amount of 30 μg of glycerol required for centrifugation. Moreover, since there is no significant difference between 2 and 3 h centrifugation, the lesser time was optimal in all cases.

**Fig. 4:**
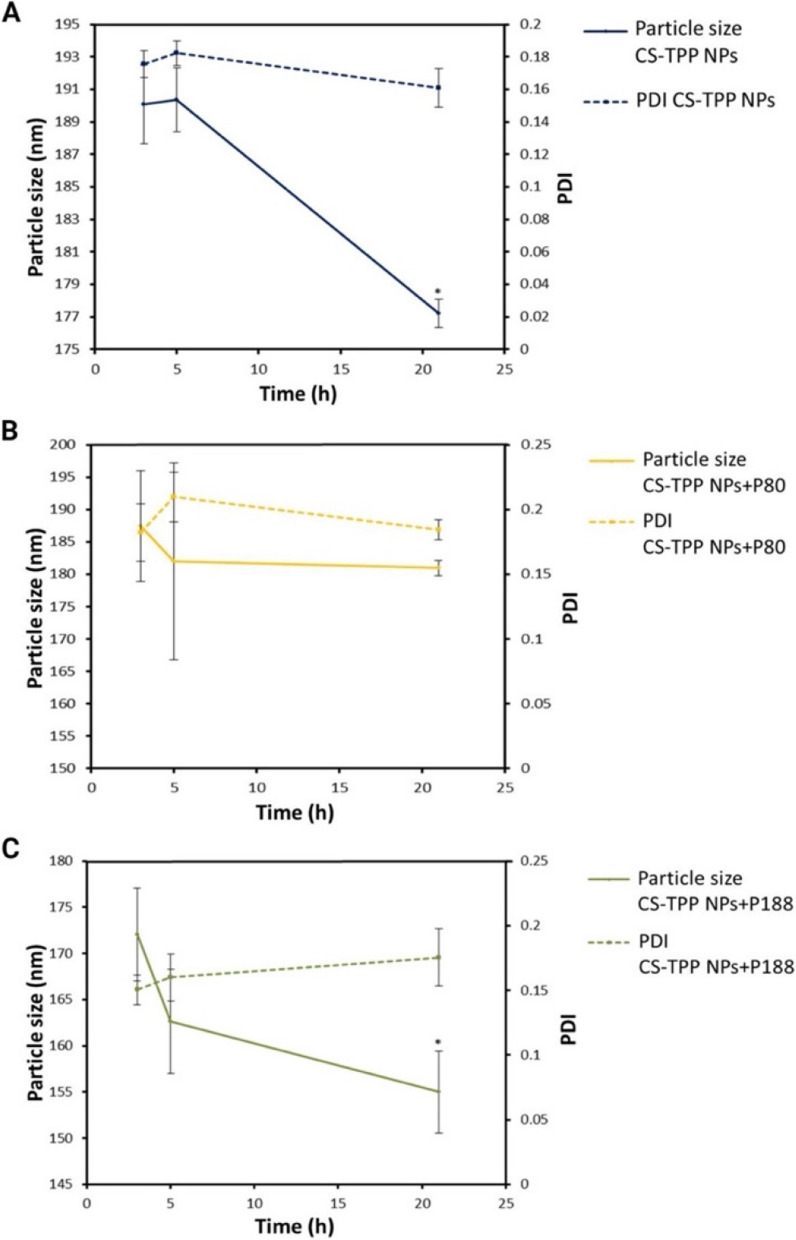
Particle size and PDI of samples as a function of dialysis time (3, 5, and 21 h). **A** CS-TPP NPs without surfactant, (**B**) CS-TPP NPs with P80, and (**C**) CS-TPP NPs with P188. *n* = 3; one-way ANOVA, Tuckey post hoc test. * *p* < 0.05 between the points studied and the sample at 3 h of dialysis

On the other hand, dialysis may offer an alternative to a mild purification method for NPs. The molecular weight cut-off size of the dialysis bag (50 kDa) is adequate to allow the outflow of excess excipients, but no outflow of the formed CS-TPP NPs occurs [38]. Figure 4 illustrates the results obtained for particle size and PDI as a function of time for the CS-TPP NPs prepared with and without surfactants. This figure indicates a decrease in the two variables studied and the standard deviation at 21 h, selecting this time to analyze the purification process.

**Fig. 5:**
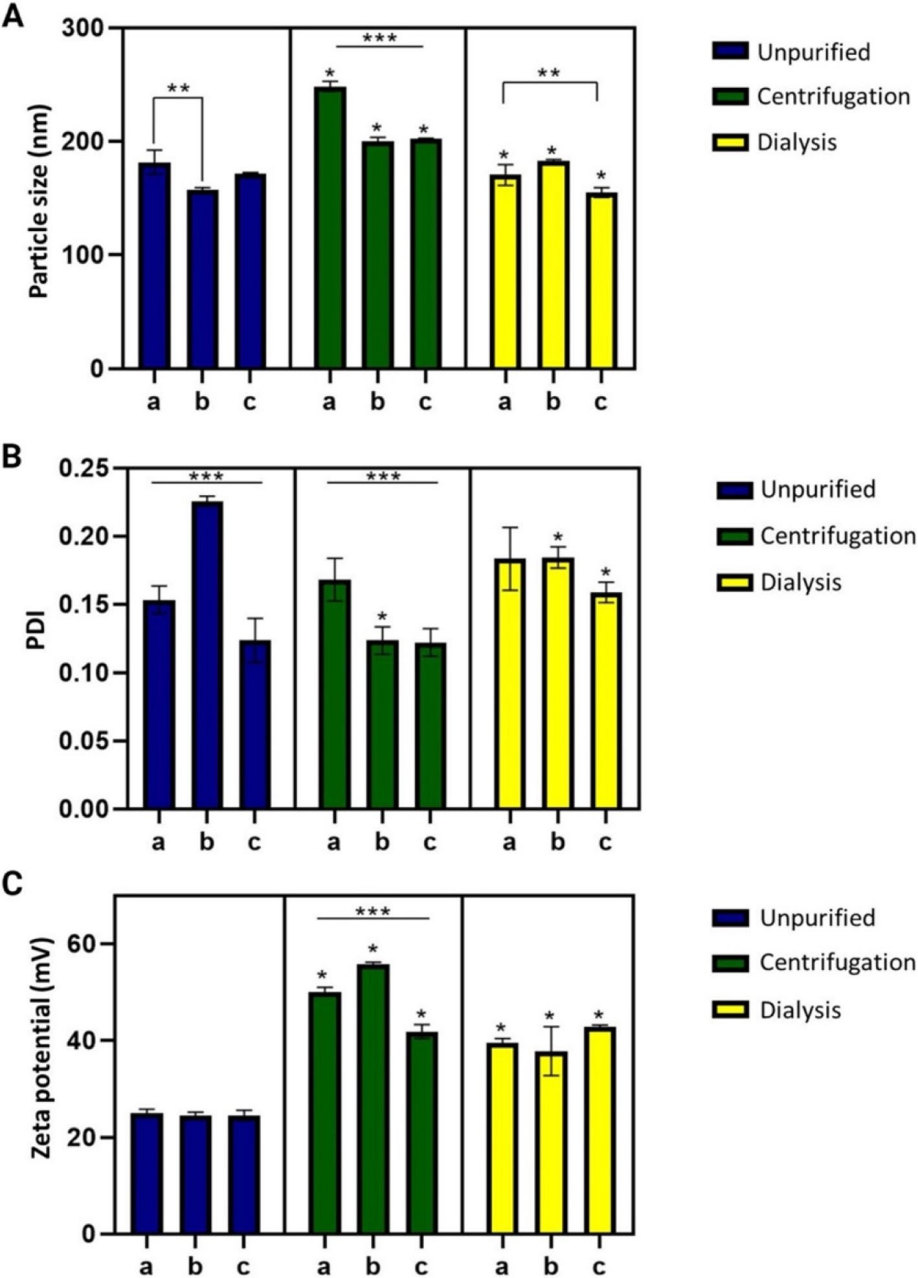
Comparison of the purification process by centrifugation and dialysis techniques; between samples with (a) CS-TPP NPs without surfactant, (b) CS-TPP NPs with P80, and (c) CS-TPP NPs with P188. **﻿A** Particle size, (**B**) PDI, and (**C**) Zeta potential. *n* = 3; two-way ANOVA, Tuckey post hoc test. * *p* < 0.05 between purified and unpurified samples, ** *p* < 0.05 between two samples, *** *p* < 0.05 between total samples of the same purification method

We evaluated the addition of two nonionic surfactants (separately), P80 and P188, to improve suspension stability and avoid the aggregation of CS-TPP NPs during purification. The centrifugation process led to the fusion of the CS-TPP NPs in all formulations, as presented in Fig. 5A, indicating the most significant increase in size in the formulation without any surfactant. Dialysis did not affect particle size in any case. The centrifugation also impacted polydispersity in CS-TPP NPs without surfactant and CS-TPP NPs with P188, although not significantly. In the case of CS-TPP NPs with P80, there was a significant decrease in PDI after this process (Fig. 5B).

Figure 5B shows that dialysis also elevated PDI in CS-TPP NPs without surfactant and CS-TPP NPs with P188, but with no significant difference due to the variability of the results. Furthermore, as in centrifugation, PDI of the CS-TPP NPs with P80 decreased after dialysis.

Figure 5C reveals a notable increase in the zeta potential after centrifugation and dialysis processes. However, this increase was less robust in CS-TPP NPs containing P80 purified by dialysis.

Regarding yields, the centrifugation process generated a higher loss of the CS-TPP NPs synthesized than the dialyzed samples, as shown in Table 4.

**Table 4:** Performance in nanoparticle synthesis under the influence of the purification method. *n* = 3; mean ± SD

Formulation	Purification method	Yield (%)
CS-TPP NPs + P80	Centrifugation	13.09 ± 1.68
	Dialysis	62.28 ± 6.21
CS-TPP NPs + P188	Centrifugation	8.28 ± 1.67
	Dialysis	70.24 ± 11.79

*n* = 3; mean ± SD

### Freeze-drying

**Fig. 6:**
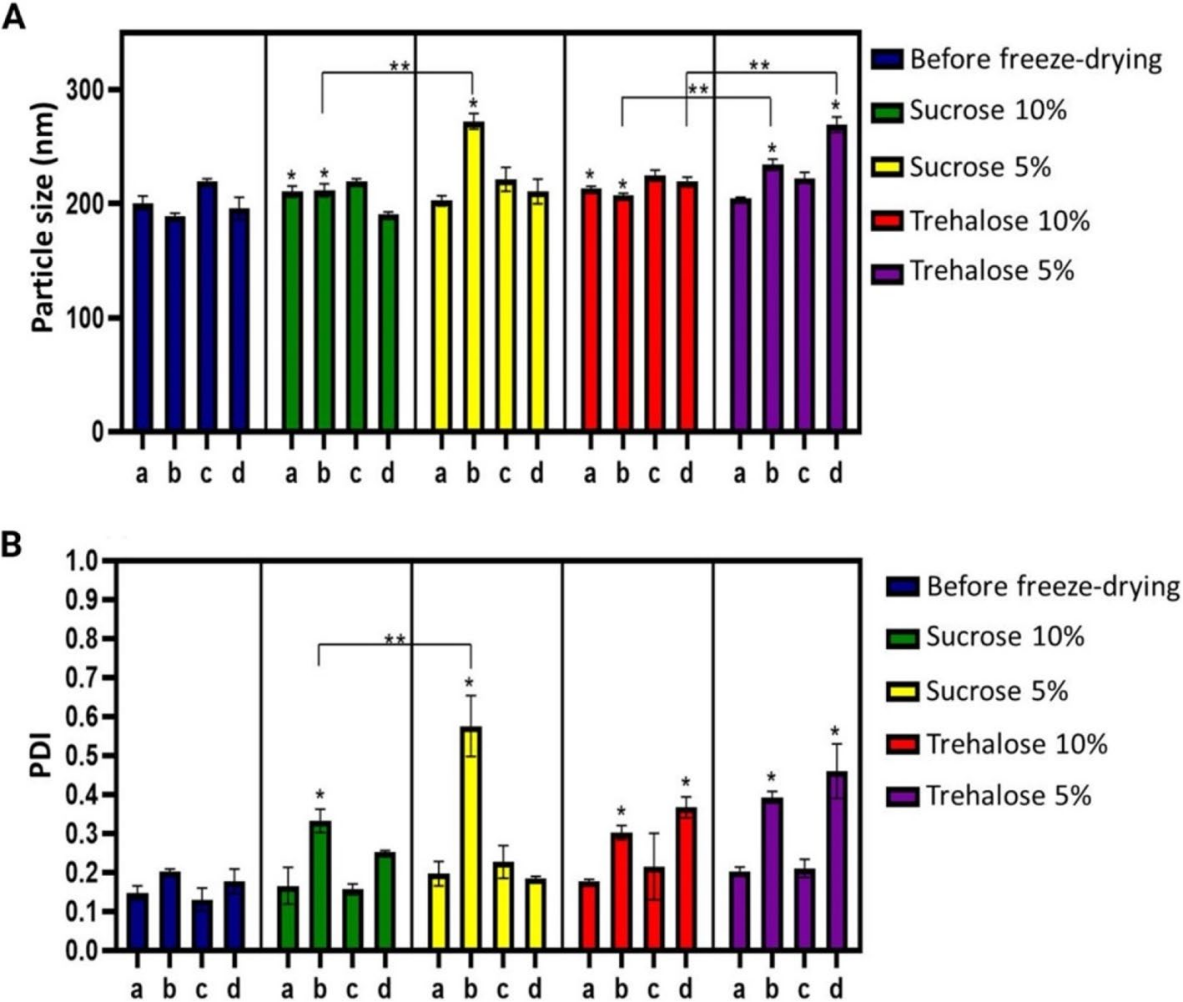
Comparison of the freeze-drying process with two cryoprotectants (sucrose and trehalose) at 5% and 10% w/v concentrations between samples of (a) CS-TPP NPs with P80 purified by centrifugation, (b) CS-TPP NPs with P80 purified by dialysis, (c) CS-TPP NPs with P188 purified by centrifugation, and (c) CS-TPP NPs with P188 purified by dialysis. **A** Particle size and (**B**) PDI. *n* = 3; two-way ANOVA, Tuckey's post hoc test. * *p* < 0.05 between freeze-dried samples and before freeze-drying. ** *p* < 0.05 between two samples

We freeze-dried the CS-TPP NPs after the purification process, except for those that did not contain surfactant due to their coalescence. We evaluated the impact of the purification processes on lyophilization, finding that none of the samples exhibited re-dispersibility; for this reason, we added two cryoprotective agents, trehalose and sucrose. Figure 6 confirms that, in general, both sugars effectively protected CS-TPP NPs during freeze-drying. However, Fig. 6A indicates a significant increase in size after freeze-drying, mainly in dialyzed samples. This behavior is more clearly appreciated in Fig. 6B, with an increased polydispersity for most of the samples that underwent the dialysis process. In almost all cases, it was sufficient to add 5% of the cryoprotectant; however, for dialyzed samples, there is a need to increase the amount of cryoprotectant to protect the particles.

### Scanning Electron Microscopy (SEM)

We conducted microscopic characterization on samples we had previously synthesized in the presence of P188 and purified through dialysis. This choice was based on the acceptable physical properties and the superior yields achieved at the conclusion of the dialysis process.

**Fig. 7:**
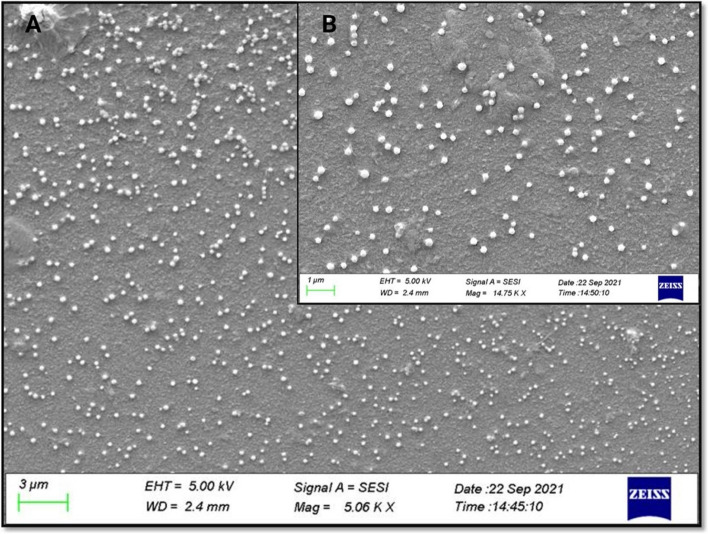
SEM images obtained from CS-TPP NPs with P80. At a magnification of (**A**) 5.06 KX and (**B**) 14.75 KX, the scale of the measuring bar is equivalent to 3 μm and 1 μm, respectively

SEM recreated a surface image of the NPs, obtaining their dimensions and general shape. In agreement with the particle size and zeta potential evaluations, the CS-TPP NPs demonstrated a spherical shape and size of approximately 200 nm with no agglomerations (Fig. 7).

## Discussion

### Optimization of parameters for synthesis

CS is an alkaline polysaccharide that is cross-linked under acidic conditions. The colloidal system of CS-TPP NPs is thermodynamically unstable, especially under unfavorable solution pH conditions and at high particle concentrations, due to the high surface energy associated with nanoscale dimensions [39]. For this reason, several parameters for optimizing CS-TPP NPs synthesis were evaluated. We discarded those samples that presented aggregates and high PDI values because it indicated a lack of stability to form the colloidal system with the NPs.

Previous research has already reported an increase in particle size as a function of increasing pH [16, 26, 40–43], which is attributed to particle aggregation resulting from a reduced repulsive potential on the surface of suspended CS-TPP NPs due to increasing pH in solution. This behavior could also explain the results obtained during our experimentation since Table 2 indicates a reduction in zeta potential when pH increases, probably due to the decrease of NH_3_^+^ groups [39]. However, this could imply an increase in the polydispersity of CS-TPP NPs [44, 45], not reflected in our results.

Most of the research that describes an increase in size with pH also indicates an increase in PDI. Therefore, it is essential to analyze the impact of all the factors that intervene in the system to explain the results of this work.

First, the smaller particle size is present at lower pH rather than solely attributed to the surface charge that generates fewer agglomerations. This behavior could be due to the extended conformation of the CS chain, which occurs mainly because of the high electron density of the protonated groups and, consequently, more accessible to the anionic TPP, generating a folding on itself (mainly through intramolecular bonds). This folding triggers more interconnected particles with increased internal cross-linking and, therefore, more compacted, causing a decrease in size [23, 41]. On the other hand, the presence of protonated amino groups decreases with increasing pH. Due to the size of the hydroxide ions, they facilitate the interaction with the internal protonated groups of the CS chain [46], leaving the NH_3_^+^ groups more external or separated, being accessible to the P_3_O_10_^−5^ and generating networks with longer chains, which leads to the increase of the particle size. Moreover, as mentioned above, the decrease in pH increases the zeta potential that at least electrostatic surface charges can lead to higher stability of the particles generating less aggregation [39]. However, this possible advantage in more acidic media can interconnect the CS-TPP NPs when there is an excess of TPP on the surface. As a result, the P_3_O_10_^−5^ ions of the TPP are more available to interact with the free protonated amino groups on the surface, as compared to a higher pH where there are fewer protonated groups. The increase in interconnections did not impact the average particle size, which indicates that there are populations with extreme dimensions, creating a wide distribution of particle sizes, which is reflected in the increase in PDI.

The appearance of the solution changed from clear to opalescent when a certain amount of TPP ions was added to the CS solution, which indicated a change in the physical states of CS to form NPs, then microparticles, and eventually aggregates [47]. In this way, the appearance of opalescence indicates the more efficient formation of NPs [23]. Based on the appearance and the optimal results obtained in both variables (Table 3), the parameters selected to elaborate the CS-TPP NPs were 500 rpm, with two minutes of dripping (0.85 mL/min), at 1% w/v of CS and TPP, at pH 5.5.

### Evaluation of surfactant addition in the formulation

The surfactant plays a crucial role in preparing the nanospheres and significantly affects their morphology. The addition of P80 in the CS solution allowed the polymer to be adequately dispersed by intercalating between the molecules to facilitate the ionic cross-linking reaction, generating a decrease in particle size, since by improving the contact with TPP, more internal interactions occur within the polymer chain, forming more compact particles [26]. Several research groups reported this behavior when adding low surfactant concentrations [28, 31, 32]; however, this decrease in particle size does not occur uniformly, directly impacting the PDI. P188 acts as a stabilizing agent during particle formation, reducing the surface energy and inhibiting particle growth, generating a monodisperse NPs solution related to the adsorption of the surfactant on the surface of the NPs, decreasing adhesion and aggregation of the CS [48, 49].

Figure 5 demonstrates the stabilizing effect of surfactants, showing that, in the particular case of P80, the polydispersity of the samples decreased after the centrifugation process. It results from forming smaller particles, compared to the average when the surfactant was employed during the synthesis of CS-TPP NPs, which generated a higher polydispersity in the initial sample. However, when centrifuged, smaller particles could remain in the supernatant.

### Purification

On the other hand, the increase in zeta potential after the centrifugation process probably reflects the presence of an excess of free TPP around the CS-TPP NPs at the end of their formation, thus masking the surface charge, but after removing the excess free NH_3_^+^ on the surface of the CS-TPP NPs remains exposed. We observed the same behavior after dialysis, again attributable to the removal of TPP, as well as to the rearrangement of the CS chains in the primary groups and efficient distribution of the cross-linked TPP anionic charges leading to the formation of more compact particles with smaller size, as suggested by Hashad et al. [20]. However, the formulations with P80 presented an increase in particle size, which could be related to particle size evolution during dialysis caused by the reorganization of intermolecular bonds and the free polymeric chains' interactions with particle network syneresis swelling [23]. Therefore, size change results from the balance between the previously mentioned forces and the fact that there is a change in the medium where the CS-TPP NPs reside. All this, in turn, also impacts the polydispersity of the particles.

Although, in general terms, dialysis is considered a simple technique, there are several factors to evaluate and control to maintain sufficient osmotic conditions, such as the medium and volume, agitation speed, and membrane cut-off [19]. These issues substantially impact this technique's success, and a slight imbalance can generate agglomerations in the system. After dialysis, the decrease in the PDI of the CS-TPP NPs with P80 may be due to the removal of smaller-sized CS-TPP NPs and rearrangement of individual and clustered NPs, generating particles with greater homogeneity in size.

### Freeze-drying

In general, polymeric NPs tend to coalesce and show aggregation after synthesis when stored for a long time, and those generated by the ionic gelation method are no exception. Along with the restricted physicochemical stability of CS-TPP NPs suspensions, the freeze-drying process may cause an irreversible aggregation of the NPs because inter- and intramolecular hydrogen bonds form during freezing and drying [24]. Furthermore, ice crystallization may induce mechanical stresses in the NPs, destabilizing them [23]. For these reasons, as expected, CS-TPP NPs could not be resuspended after the freeze-drying process. As reported, stabilizers such as P188 and P80 within the formulation could preserve the NPs viability after lyophilization [50]. However, this did not occur in the treated samples, probably due to the stabilizers' low concentration and the CS's lability.

Therefore, we evaluated the addition of two cryoprotectants, trehalose and sucrose. Both stabilized the NPs, facilitating their redispersion and preserving their size in most cases. This protective interaction was possible due to the ability of the sugars to remain amorphous during freeze-drying, interacting with the solute through hydrogen bonds, which keeps the solute in a "pseudo hydrated" state during the dehydration stage protecting it against damage during dehydration and subsequent rehydration [23]. Also, increasing the amount of cryoprotectant was necessary to stabilize the particles that previously went through the dialysis process for purification. A possible explanation is that in terms of percentage concentration by weight, the centrifuged samples have a higher amount of cryoprotectants than the dialyzed ones since the first ones have a lower amount of solids per sample volume due to the increased loss of CS-TPP NPs during centrifugation, Table 4.

It has been reported the superiority of trehalose in preserving the structure of NPs after lyophilization compared to several sugars, including sucrose. This attribute could be related to its peculiar characteristics: low hygroscopicity, the absence of internal hydrogen bonds (which allows a more flexible formation of hydrogen bonds with NPs), very low chemical reactivity, and a higher glass transition temperature Tg [23, 51]. Although no literature explains the equivalence in the results between trehalose and sucrose, there is evidence that shows a similar behavior of the results with the present study [24]. It is also important to consider the possible protective effect of the stabilizers present in the formulation during the freeze-drying process. Although it was impossible to appreciate this effect on their own, the combination could be favorable because the sugar dehydrates the surfactant in the bulk solution, forcing it to the particle surface (to stabilize the particles). As the unfrozen water is removed during drying, according to the water replacement hypothesis, the cryoprotectants serve as water substitutes to form bonds with the stabilizers on the surface of the NPs; thus, these will not aggregate at the end of drying [52]. Additionally, forming a hydrophilic layer by the stabilizer on the particle surface helps redispersion after freeze drying [53, 54].

#### SEM

The images generated by SEM allowed observing adequate structures following the idea that the smaller size NPs decrease the delivery distance, facilitating the penetration of the NPs until they reach the target cells where they would have their activity [6], in addition to favoring the antimicrobial activity provided by the CS [26]. Another critical point to consider is the shape, which highly influences the possible toxicity of the nanomaterial since spherical shapes generate less harmful effects on tissues [55, 56].

## Conclusion

Beyond having achieved acceptable physical characteristics for the transporters under mild conditions and having a methodology that is easy to implement, it is of utmost importance to understand the interactions present in the formulation during the evaluation of the synthesis, purification and conservation parameters of the CS-TPP NPs. This knowledge is essential to further optimize the formulation and obtain systems with suitable properties that allow their application in the biomedical field.

Based on the factors analyzed in this study, the optimal synthesis conditions are as follows: a concentration of 0.1% of both TPP and CS, with a pH of 5.5 and a drip time of 2 min at 500 rpm. This resulted in an average particle size of 172.8 ± 3.937 nm, a PDI of 0.166 ± 0.008 and a zeta potential of 25.00 ± 0.79 mV. Evaluation of the results suggests the possibility of adjusting the amount of TPP added to improve the stability of the system. Future studies with sensitive molecules loaded in the system are needed to demonstrate the preservation of their properties and the preservation of the properties of the active principle.

On the other hand, the purification process induced an increase in the zeta potential, reaching values of 40.8–56.2 mV by centrifugation and 32.0–43.2 mV by dialysis. However, it failed to prevent particle fusion, especially in centrifugation, and increased polydispersity in dialysis. These effects were reduced by the addition of surfactants (P80 and P188). Although the dialysis technique for NPs purification is not as common as centrifugation, it is interesting to consider its advantages in terms of final yields. With regard to the choice of surfactants, it is crucial to consider the benefits they offer to the formulation. For this, it is necessary to carry out further studies on their interaction with the molecule to be loaded in the NPs and even to evaluate the combination of both surfactants.

With respect to lyophilization, both sucrose and trehalose showed an acceptable protective effect, with a concentration of 5% w/v being sufficient to preserve the physical properties without statistically significant changes. However, an adjustment of the w/w concentration should be considered if it is desired to work with dialysis-purified NPs.

## Data Availability

The datasets used and/or analysed during the current study available from the corresponding author on reasonable request.
